# Recurring DNA copy number gain at chromosome 9p13 plays a role in the activation of multiple candidate oncogenes in progressing oral premalignant lesions

**DOI:** 10.1002/cam4.307

**Published:** 2014-07-24

**Authors:** Rebecca Towle, Ivy F L Tsui, Yuqi Zhu, Sara MacLellan, Catherine F Poh, Cathie Garnis

**Affiliations:** 1 Department of Integrative Oncology, British Columbia Cancer Research CentreVancouver, BC, Canada; 2 Department of Pathology and Laboratory Medicine, University of British ColumbiaVancouver, BC, Canada; 3 Oral Biological and Medical Sciences, University of British ColumbiaVancouver, BC, Canada; 4 Division of Otolaryngology, Department of Surgery, University of British ColumbiaVancouver, BC, Canada

**Keywords:** 9p13, copy number gain, dysplasia, head and neck cancer, oncogene, oral squamous cell carcinoma, overexpression, premalignant lesion, progression

## Abstract

Genomic alteration at chromosome 9p has been previously reported as a frequent and critical event in oral premalignancy. While this alteration is typically reported as a loss driven by selection for *CDKN2A* deactivation (at 9p21.3), we detect a recurrent DNA copy number gain of ∼2.49 Mbp at chromosome 9p13 in oral premalignant lesions (OPLs) that later progressed to invasive lesions. This recurrent alteration event has been validated using fluorescence in situ hybridization in an independent set of OPLs. Analysis of publicly available gene expression datasets aided in identifying three oncogene candidates that may have driven selection for DNA copy number increases in this region (*VCP*, *DCTN3*, and *STOML2*). We performed in vitro silencing and activation experiments for each of these genes in oral cancer cell lines and found that each gene is independently capable of upregulating proliferation and anchorage-independent growth. We next analyzed the activity of each of these genes in biopsies of varying histological grades that were obtained from a diseased oral tissue field in a single patient, finding further molecular evidence of parallel activation of *VCP*, *DCTN3*, and *STOML2* during progression from normal healthy tissue to invasive oral carcinoma. Our results support the conclusion that DNA gain at 9p13 is important to the earliest stages of oral tumorigenesis and that this alteration event likely contributes to the activation of multiple oncogene candidates capable of governing oral cancer phenotypes.

## Introduction

Genomic instability, a hallmark of most epithelial tumors, can drive activation of oncogenes and silencing of tumor suppressor genes via changes in DNA dosage 1,2. While many critical DNA amplifications and deletions have been uncovered for invasive tumor tissues, few genomic studies of earlier precancerous lesions have been undertaken. This represents a missed opportunity to identify the molecular origins of disease, as causal genetic alterations are more likely to be discovered through analysis of premalignant tissues.

Oral cancers contribute significantly to the global cancer problem, accounting for tens of thousands of deaths worldwide each year 3. Efforts to uncover early genetic changes have been previously undertaken on oral premalignant lesions (OPLs). To date, few molecular analyses of OPLs have identified genetic markers to help determine the risk of progression from premalignancy to invasive cancer 4–11. Loss of chromosome 9p has been reported as both a recurring event and an alteration predictive of increased risk of disease progression in OPLs 5,6,12,13. However, these studies have typically relied on loss of heterozygosity (LOH) analysis of only a small number of microsatellite markers on chromosome 9p. Furthermore, these studies often excluded analyses of lower grade OPLs (mild/moderate dysplasia), which is necessary to glean insights into disease initiating molecular changes.

We have previously reported that the degree of global genomic dysregulation is associated with progression risk for low-grade OPLs 4. Here we report a DNA gain at 9p13 that recurs in low-grade OPLs that subsequently progress to invasive oral cancer, yet is absent in nonprogressing low-grade OPLs. Significantly, this alteration is a more frequent event in progressing low-grade OPLs than canonical loss of chromosome 9p. Also, we provide functional data indicating that this DNA gain spans three potential oncogenes in oral carcinogenesis. This finding suggests that a single, focal DNA copy number gain may be dysregulating multiple genes in concert in order to drive progression to invasive oral cancer.

## Materials and Methods

### Whole genome characterization of DNA copy number alterations in OPLs

This study evaluated 64 OPLs (43 high-grade dysplasias, 7 low-grade dysplasias that later progressed to invasive disease, and 14 low-grade dysplasias that did not progress) for DNA copy number alteration status on chromosome 9p. Previously described whole genome tiling-path array comparative genomic hybridization (CGH) data were used to perform this analysis 9. All profiles have been deposited to NCBI Gene Expression Omnibus (GEO), series accession number GSE9193.

### Gene expression profiling analysis

Expression of the 58 genes mapped within the recurring 9p13.3 DNA gain was evaluated using previously published data with the gene expression profiles of 10 tumor samples and their paired adjacent normal tissue (GEO accession number GSE46802) 14.

### Accrual of fresh oral tumor tissues for DNA copy number and gene expression analyses

Four fresh frozen tissues were obtained immediately following surgical resection in the operating room 15. Collected tissues were microdissected based on pathologist guidance. Extracted DNA was hybridized to a whole genome tiling-path CGH microarray 16,17 and corresponding extracted RNA was analyzed by Agilent Whole Human Genome Microarray 4 × 44K (Agilent Technologies, Mississauga, Ontario, Canada). Sample labeling, hybridization, and scanning of the tiling-path CGH arrays was performed as described previously 9,17. Labeling and hybridization experiments for the Agilent 4 × 44K array—which interrogates >41,000 unique human transcripts—were performed according to manufacturer's protocols. These gene expression arrays were scanned using Axon GenePix 4000B and 4200A scanners. Median normalization was performed on Agilent Whole Human Genome Microarray as described previously 18. All data are publicly available on GEO (GSE46802).

### Statistical analysis of genomic profiles

A three-step normalization procedure was used to remove systematic biases on tiling-path CGH arrays as described previously 19,20. *SeeGH* software was used to display log_2_ signal intensity ratios in relation to genomic locations in the hg17 assembly (NCBI Build 35) 19. Data points with standard deviation >0.075 and signal-to-noise ratio <3 in either channel were filtered from downstream analyses.

### Validation of gene candidates in tissue microarrays

An independent validation set of samples consisting of premalignant archival patient tissues from the British Columbia Oral Biopsy Service were used to construct a tissue microarray (TMA). Thirty-seven mild and moderate dysplasia cases with known progression status and 26 severe dysplasia and carcinoma in situ (CIS) specimens were used for assembly of the TMA (all cases were formalin fixed and paraffin embedded [FFPE]). Patient demographic information is listed in Table S1. Briefly, one 1-mm core from a represented area was obtained from each paraffin specimen and distributed on recipient TMA blocks using a specific arraying device (Manual Tissue Arrayer MTA-1; Beecher Instruments, Inc., Sun Prairie, WI). A single 5-*μ*m section was then cut from the TMA block and used for in situ hybridization analysis as described previously 11. One set of three-colored probes (Vysis, Downers Grove, IL) was performed on the 5-*μ*m tissue sections according to manufacturer's instructions, which included CEP9 probe (centromere, SpectrumGreen), 9p13 probe (SpectrumAqua), and 9p21 probe (SpectrumOrange). Signals were captured and imaged using Olympus BX61 (Olympus America, Inc., Melville, NY) and ImagePro Plus 5.1 (Media Cybernetics, Silver Spring, MD). At least 150 nonoverlapping intact nuclei were scored. Samples with >90% nuclei showing signals were considered informative. Sample signals were scored and classified as deletions if >50% of nuclei showed ≤1 signal and as gained or amplified if >10% of nuclei showing ≥4 signals 21.

### Cell culture and reagents

Six oral cancer cell lines (SCC-4, SCC-9, SCC-15, SCC-25, A253, and Cal27) were purchased from the American Type Culture Collection (ATCC). The oral dysplasia cell line POE9n-tert was purchased from the Harvard Skin Disease Research Center Cell Culture Core. DNA was analyzed using whole genome tiling-path CGH arrays and RNA was analyzed by Agilent Whole Human Genome Microarray 4 × 44K. SCC-9 and Cal-27 tongue cell lines were chosen for knockdown and overexpression experiments due to their genomic and expression profiles 18. They were maintained according to distributor recommendations. POE9n-tert, a premalignant oral mucosal keratinocyte cell line, was chosen for overexpression experiments due to its genomic and expression profile and was grown using keratinocyte serum-free medium supplemented with l-glutamine, bovine pituitary extract, and epidermal growth factor (Life Technologies, Gaithersburg, MD) and grown at 37°C. Relative gene expression data for each candidate gene is supplied in Table S2. 293T cells were a generous gift from Dr. Aly Karsan and were cultured in Dulbecco's Modified Eagle's medium with 10% fetal bovine serum at 37°C.

### shRNA lentiviral vector knockdown

Human pLKO.1 lentiviral shRNA target gene sets were selected from the RNAi consortium (TRC) and were purchased from Open Biosystems (Huntsville, AL). For each of the genes, five shRNAs constructs were tested for knockdown efficiency and the two that showed the best knockdown were selected for further experiments to minimize off-target effects. To produce lentivirus, 293T cells were transfected with pLKO.1 plasmid construct coding an shRNA targeted for each gene candidate with the packaging plasmids VSVG and d8.91 using TransIT-LT1 transfection reagent (Mirus, Mississauga, Ontario, Canada). pLKO.1 empty vector was also transfected into 293T cells to serve as a control. Viral supernatant was collected over 2 days post transfection, filter sterilized (0.45 *μ*m), and stored in −80°C.

After transfecting SCC-9 cells with each lentivirus for 24 h, cells were selected with 2 *μ*g/mL puromycin over 3 days. All nontransfected cells were dead within 3 days of selection, while stably transfected SCC-9 cells were effectively cultured in growth media containing 2 *μ*g/mL puromycin.

### Plasmid construction and viral transduction

Full-length human cDNA expression vectors were purchased from Open Biosystems for each gene candidate, including pCMV-SPORT6 for *VCP* (BC110913), pOTB7 for *STOML2* (BC002442), and pOTB7 for *DCTN3* (BC000319). Coding sequences were polymerase chain reaction (PCR)-amplified and cloned into pLenti4/V5-DEST Gateway® Vector as per the manufacturer's protocols (Invitrogen, Carlsbad, CA). Lentivirus supernatant was produced and collected as described above.

Twenty-four hours following infection with lentivirus Cal-27 and POE9n-tert cells were selected using 10 and 0.5 *μ*g/mL zeocin, respectively.

### Real-time PCR of mRNA expression

Total RNA from stably selected cell lines was extracted using TRIzol (Invitrogen) and treated with DNA-free DNase Treatment and Removal Reagents (Ambion, Austin, TX). High-Capacity cDNA Reverse Transcription Kit (Applied Biosystems, Foster City, CA) was used to convert total RNA to cDNA. Real-time PCR using TaqMan Universal PCR master mix was performed to analyze RNA expression levels with Applied Biosystems Standard Real-Time PCR systems. TaqMan gene expression assays of *DCTN3* (Hs00989657_m1), *STOML2* (Hs00203730_m1), *VCP* (Hs00997650_m1), and 18s rRNA (Hs99999901_s1) were purchased from Applied Biosystems. Triplicate reactions were performed for each sample and standard error was calculated using ABI software (Applied Biosystems, Foster City, CA). Relative expression values using the average of cycle thresholds of target genes and 18s rRNA were calculated using the 2^−ΔΔCt^ method. A value of 1 was assigned to the control empty vector sample.

### Western blotting

Cell lysates were harvested in radioimmunoprecipitation assay buffer (RIPA) (150 mmol/L NaCl, 1% Triton X-100, 0.1% SDS, 0.5% Na-deoxycholate, 1 mmol/L Ethylenediaminetetraacetic acid) with 10 mmol/L phosphatase cocktail I and cocktail II (Sigma-Aldrich, St. Louis, MO) and 1:100 of protease inhibitor (Invitrogen). The protein concentrations were determined using the Bicinchoninic Acid Protein assay kit (Thermo Scientific, Waltham, MA). A total of 20 *μ*g of protein was separated by NuPAGE 4–12% *Bis*-Tris Gels (Invitrogen) and transferred to polyvinylidene difluoride membranes (Millipore, Etobicoke, ON, Canada). Membrane blocking was performed in 5% w/v nonfat dry milk, 1× TBS, and 0.1% Tween-20 at room temperature with gentle shaking for 1 h for polyclonal anti-DCTN3 primary antibody (Sigma-Aldrich). Membrane blocking was performed in 5% bovine serum albumin, 1× TBS, and 0.1% Tween-20 at 4°C with gentle shaking overnight as recommended by the manufacturer before incubation with monoclonal anti-VCP (7F3) (Cell Signaling, Technology, Inc., Danvers, MA) and polyclonal anti-STOML2 (H-180) (Santa Cruz Biotechnology Inc, Santa Cruz, CA). After blocking, the membranes were incubated with the appropriate primary antibody anti-DCTN (1:1000) at 4°C with gentle shaking overnight, anti-VCP (1:1000) at 4°C with gentle shaking for 3.5 h and anti-STOML2 (1:1000) at 4°C with gentle shaking for 1 h. After washing, the membranes were incubated with peroxide-conjugated anti-mouse secondary antibody (GE Health care, Buckinghamshire, UK) and HRP-linked anti-rabbit secondary antibody (Cell Signaling, Technology, Inc.) at (1:2000) at room temperature for 45 min. Anti-*β*-actin antibody (Cell Signaling, Technology, Inc.) (1:1000) was used as loading control. Proteins were detected with the enhanced Amersham ECL Western Blotting detection kit (GE Healthcare, Buckinghamshire, UK).

### MTT cell viability assay

Stably selected cells with confirmed knockdown or overexpression efficiency were plated in five 96-well plates at a density of 1000–2000 cells per well. The number of viable cells was followed up for 5 days. Colorimetric thiazolyl blue tetrazolium bromide (Sigma-Aldrich, Oakville, ON, Canada) was added to each well (final concentration 0.5 mg/mL). For each plate, the cells were repeatedly plated in six wells and 570 nm absorbance was measured (with reference to 650 nm) using an EMax plate reader (Molecular Devices, Sunnyvale, CA). The mean of the absorbance was plotted against time and standard error of the mean was plotted as error bars. Statistical analysis was carried out using Student's *t*-test on day 5 and *P* < 0.05 was used as a cutpoint for statistical significance.

### Soft agar colony formation assay

Bottom layer agarose was made to 0.5% in a 12-well plate. The top layer was made with 2000 stably infected cells in 0.37% agarose using low-melting point agarose. The number of colonies per plate was counted for both the infected cell line and the empty vector control. All experiments were performed in triplicate wells with two biological replicates. Colonies, which consisted of ∼15 cells, were counted by two independent observers.

## Results

### Identification of recurring DNA gain at 9p13.3 in progressing OPLs

Our group previously reported that a high degree of global genomic imbalance is associated with low-grade (mild and moderate dysplasia) OPLs that subsequently progressed to invasive cancer (relative to nonprogressing low-grade OPLs) 4. Within these data, genomic imbalance at chromosome 9p was found to be the most frequently occurring event in progressing low-grade OPLs, with incidence of ∼80%. Low-grade OPLs that did not subsequently progress to invasive disease did not harbor genetic alteration at chromosome 9p13. Interestingly, genetic gain at 9p13 occurs more frequently in early OPLs than other canonical early genomic alterations for low-grade OPLs such as loss of chromosome 9p21 or chromosome 3p (78% vs. 56% and 22%, respectively).

**Figure 1:**
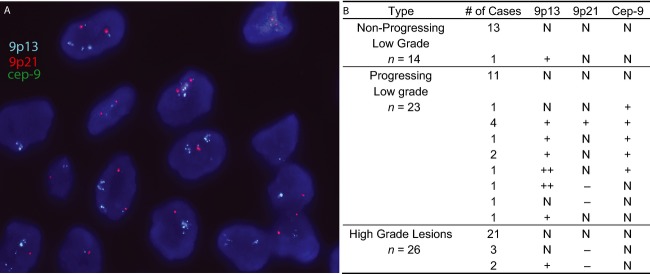
(A) Detection of *9p13* gain using FISH analysis. A representative tissue microarray of a patient with *9p13* (blue) amplification and normal copy number for *9p21* (red) and *Cep-9* (green), shown at 60× magnification. (B) *9p13*, *9p21*, and *Cep-9* FISH results of tissue microarrays for 14 nonprogressing low-grade lesion, 23 progressing low-grade lesions, and 26 high-grade lesions. *N*, normal copy number; +, gain; ++, amplification; −, deletion.

Using a TMA consisting of an independent validation set of 37 low-grade OPLs (including 23 cases that were known to later progress), we validated our findings by FISH analysis for alteration at 9p21 and 9p13 (Fig.1A–B). Among all low-grade lesions (progressing and nonprogressing), 11 cases showed a gain of *9p13* with six having normal DNA copy number at 9p21 and one exhibiting 9p21 loss. Among low-grade OPLs, 12 of 23 progressing low-grade dysplasias showed abnormal DNA copy number involving at least one analyzed locus while only one of 14 nonprogressing OPLs exhibited abnormal DNA copy number at either of these two loci (*P* = 0.01). Four (33%) of 12 progressing cases showed a gain of all three analyzed loci, suggesting a whole chromosome arm gain. Among progressing cases exhibiting DNA copy number changes by FISH, 10 of 12 cases showed gain of 9p13 while two cases showed deletion of 9p21. Analysis of demographic data for all low-grade OPLs did not show 9p13 gain correlating to age, sex, or smoking status. We also examined the frequency of 9p13 and 9p21 alteration in a panel of 26 high-grade OPLs (severe dysplasias and CIS cases) and found only two cases with gain of 9p13 and deletion of 9p21 and three cases showing no change of 9p13 but deletion of 9p21.

**Figure 2:**
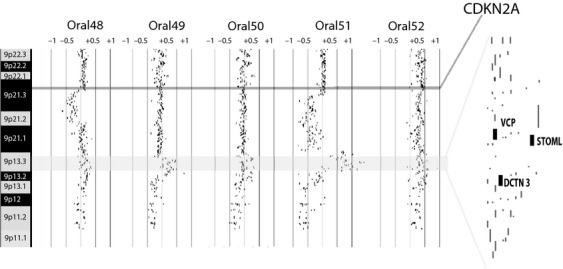
Alignment of genomic alteration data of five low-grade oral premalignant lesions (OPLs) reveals copy number gain at chromosome 9p13. Log2 signal intensity ratios of each competitive hybridization with normal reference DNA are plotted. Each black dot represents the log2 signal intensity ratio of an individual clone mapping to the array. Sample identifiers are listed at the top of genomic plots. Vertical lines represent log2 signal intensity ratios of +1, +0.5, 0, −0.5, and −1. Horizontally highlighted regions indicate the location of the CDKN2A tumor suppressor gene and the minimal altered region of the 9p13 amplicon. All known RefSeq genes within the minimal region of alteration are indicated on the right. The sizes of the three candidate genes are not drawn to scale.

Mapped more specifically, the recurrent high-level DNA copy number gain in progressing OPLs occurs at 9p13.3 (bp 33,751,040–36,241,407 [hg19]), which is 2.49 Mbp in size and spans 58 unique RefSeq genes (Fig.2). We reviewed publicly available gene expression data derived from 10 oral cancer tumors to hone this gene list and identified possible oncogenes “driving” the emergence of this recurrent gain 14. Candidate genes were selected based on (1) increased expression in tumor samples (relative to normal comparators), (2) previous implication as oncogenes in other cancer types, and (3) plausible positive impacts on tumorigenicity when overexpressed. Seven of 58 genes mapping to 9p13.3 in this dataset were found to be ≥twofold overexpressed in oral tumors in ≥6 of 10 patients. Of these, three genes have been previously implicated as being overexpressed in malignant processes: *valosin-containing protein* (*VCP*), *stomatin-like protein 2* (*STOML2*), and *dynactin 3* (*DCTN3*) 22–28. We selected these candidates for further study.

### Inhibition of gene candidates within the 9p13.3 amplicon diminishes oral cancer phenotypes

**Figure 3:**
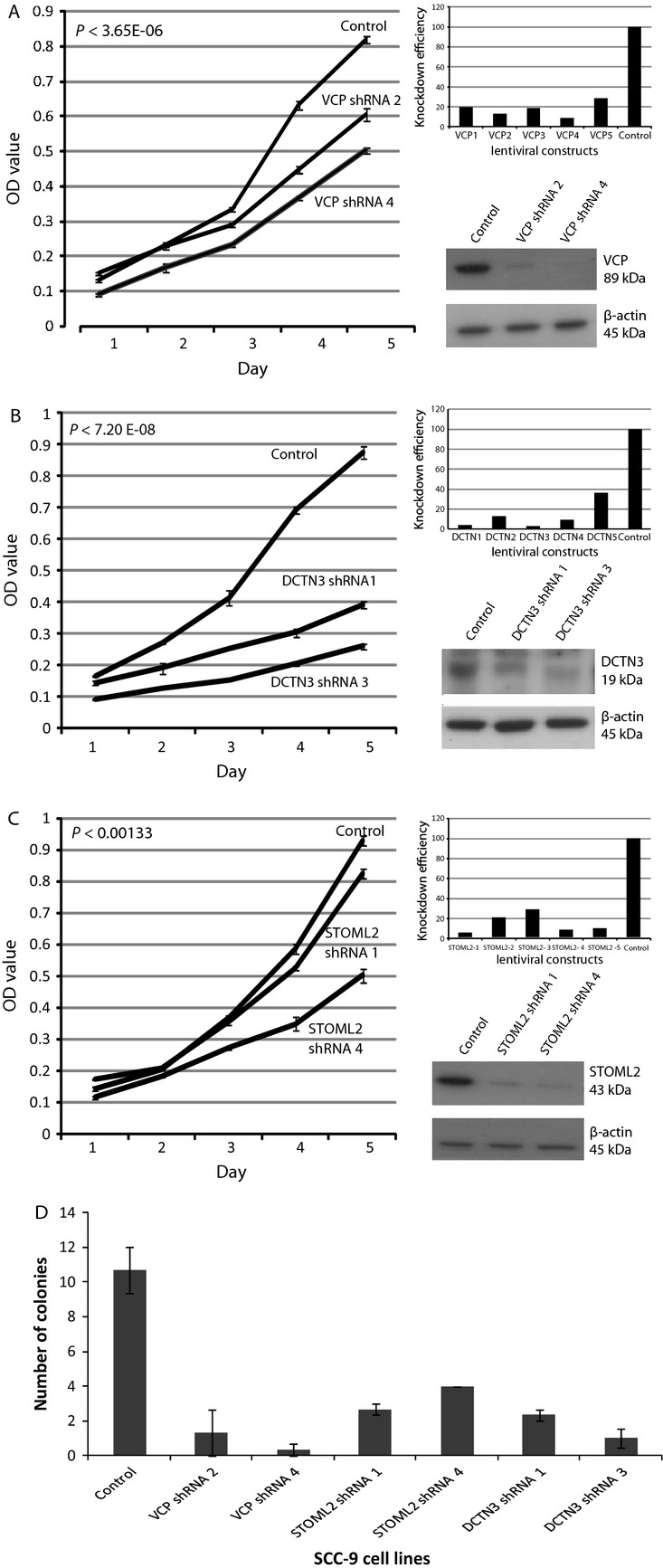
Candidate gene silencing contributes to decreased cell proliferation in SCC-9 cells (an oral cancer cell line harboring the recurrent 9p13 amplicon). (A) presents VCP, (B) DCTN3, (C) STOML2. Each panel contains three experiments performed for each gene, presenting images of, knockdown efficiencies (qRT-PCR) for five different shRNAs against the candidate gene, protein expression of two best knockdowns (Western blots), and MTT proliferation results over a 5-day period of the two confirmed knockdowns. (D) shRNA stable knockdown of each candidate gene in SCC-9 cells decreases colony formation in soft agar relative to control SCC-9 cells. The two most effective shRNAs for each candidate gene are shown. Triplicate experiments were performed for each line. The mean number of colonies and standard deviations are plotted.

The functional significance of *VCP*, *STOML2*, and *DCTN3* in oral cancer phenotypes was evaluated by shRNA-mediated knockdown experiments using oral cancer cell line SCC-9. SCC-9 cells were chosen due to the existence of high-level amplification of the 9p13 region (bp 34,085,033–35,428,177 to bp 35,432,063–36,539,166), which corresponds to the 9p13 DNA gain we observed in clinical samples 18. To minimize off-target effects, the knockdown efficiencies of five shRNAs for each gene were tested. The two shRNAs demonstrating the most effective knockdown were used for further experiments. The reduced expressions of candidate genes were confirmed at both transcript and protein levels (Fig.3). In summary, our results indicated that knockdown of each of the three candidate genes resulted in reduced cell proliferation rates, with inhibition of VCP and DCTN3 causing the most dramatic reductions in SCC-9 cell growth (Fig.3A–D). Additionally, independent knockdown of each candidate gene in SCC-9 cells also decreased anchorage-independent growth in soft agar (Fig.3D).

We also attempted to knockdown these candidate genes in the cell line dysplastic oral keratinocyte, an oral dysplasia cell line that also possesses a DNA gain at 9p13. However, in multiple experiments, the loss of the candidate gene resulted in the death of cells before any additional experiments could be performed. The vector control cells remained viable.

### Overexpression of candidate genes enhances cell proliferation and anchorage-independent growth

**Figure 4:**
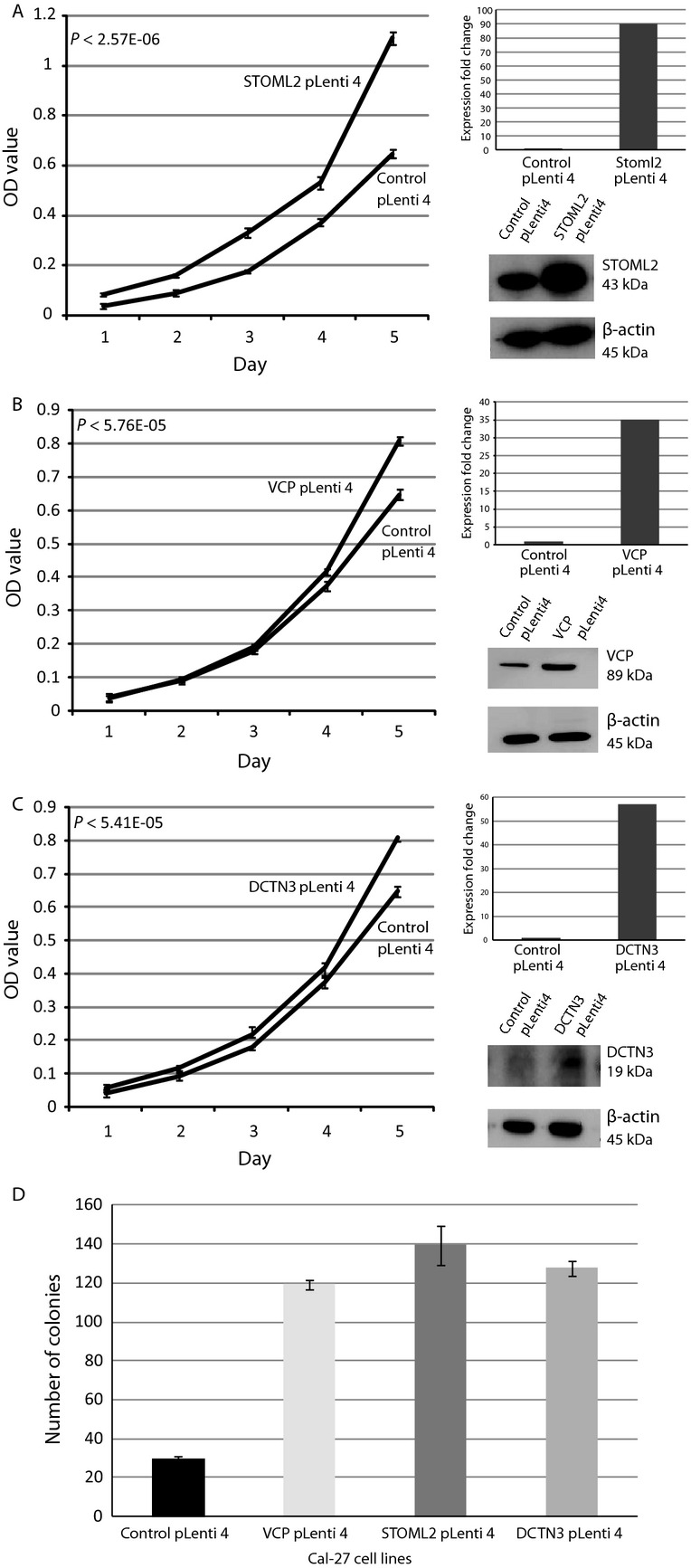
Stable overexpression of STOML2 (A), VCP (B), and DCTN3 (C) increases cell growth in Cal-27 cells (an oral cancer cell line not exhibiting the 9p13 amplicon). MTT proliferation results over a 5-day period are shown. Each panel for each gene depicts (1) an MTT cell proliferation plot for both control and test lines, (2) qRT-PCR results demonstrating increased expression of a given candidate gene in test line, and (3) Western blots confirming concordant protein overexpression of test line. (D) Stable overexpression of candidate gene in Cal-27 cells increases colony growth in soft agar relative to control Cal-27 cells. Experiments were performed in triplicate. Mean number of colonies and standard deviations are plotted.

In evaluating the ability of each gene candidate to promote growth in oral cancer, we focused on cell line Cal-27, an oral cancer cell line that is DNA copy number neutral at chromosome 9p13. We created Cal-27 sublines stably overexpressing each gene candidate using lentiviral constructs. Overexpression of each gene candidate in the stable clone was verified by qRT-PCR and Western blotting (Fig.4). Overexpression of VCP—measured as a 35-fold mRNA increase relative to control (empty vector) infected Cal-27 cells—resulted in the most significant increase in cell proliferation (*P* = 5.7 × 10^−5^; Fig.4 A–D). VCP-expressing cells also showed a dramatic increase in the number of colonies formed in soft agar and colony size compared to vector control cells (Fig.4D). Increased colony formation was also observed in STOML2- and DCTN3-overexpressing cells, indicating that these candidate genes have a role in enhancing anchorage-independent growth as well.

**Figure 5:**
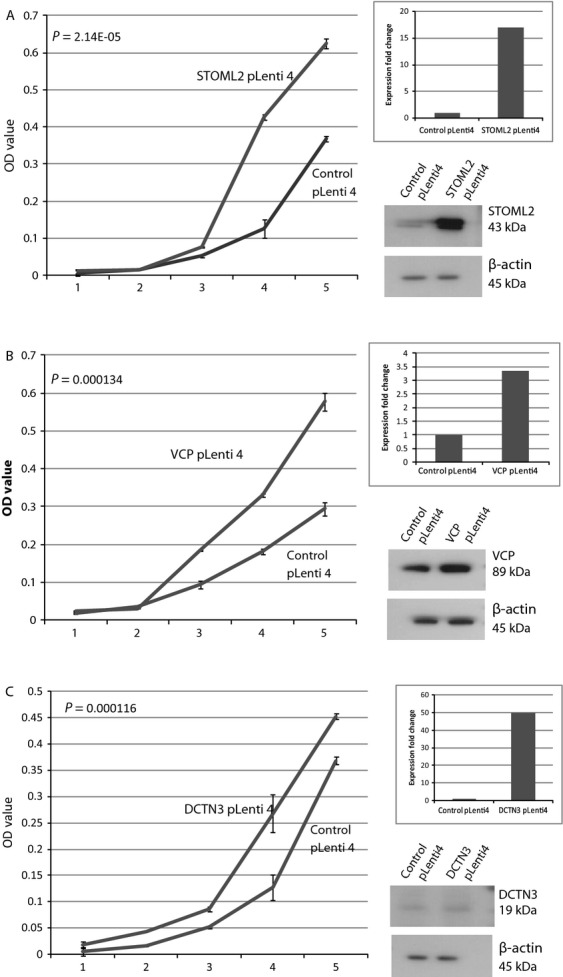
Stable overexpression of STOML2 (A), VCP (B), and DCTN3 (C) increases cell growth in the dysplasia cells line, POE9n-TERT (an oral dysplasia cell line with no 9p13 amplicon). MTT proliferation results over a 5-day period are shown. Each panel for each gene depicts (1) an MTT cell proliferation plot for both control and test lines, (2) qRT-PCR results demonstrating increased expression of a given candidate gene in test line, and (3) Western blots confirming increased amount of protein of test line.

We also assessed the ability of the candidate genes to affect proliferation in premalignant cells. We overexpressed each candidate gene in POE9n-tert, an oral dysplasia cell line that does not possess the 9p13 DNA copy number gain. As with Cal-27 tumor cell lines, each candidate gene caused a significant increase in the proliferation of the candidate gene expressing cell lines compared to the empty vector control cell line (*VCP*: *P* = 1.34 × 10^−4^, *DCTN3*: *P* = 1.16 × 10^−4^, *STOML2*: *P* = 2.14 × 10^−5^; Fig.5).

### 9p13 amplification within multiple biopsies from a single patient

**Figure 6:**
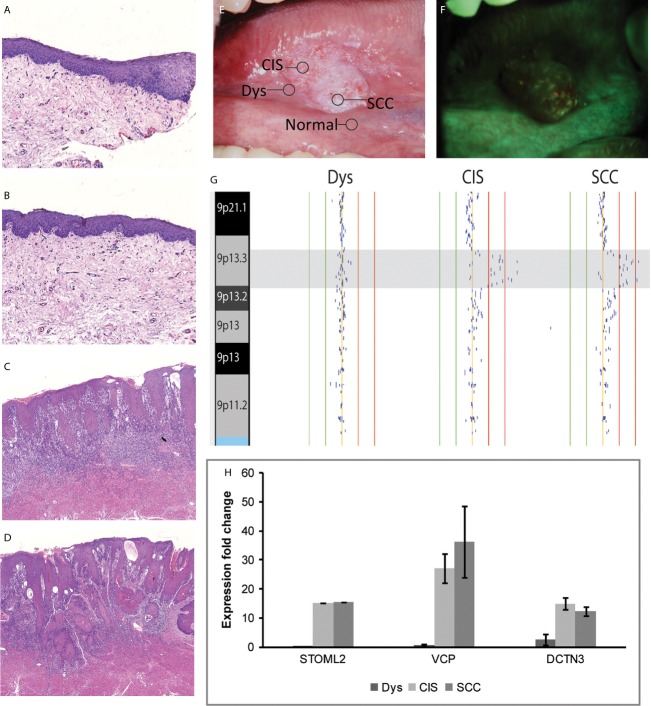
DNA gain at 9p13 and corresponding increased mRNA expressions in gene candidates detected in biopsies from a single patient. Clinical, pathological, and molecular characterization of different tissues obtained from an oral cancer disease field in a single patient. (A) to (D) are photomicrographs of hematoxylin and eosin stained slides of varying histology: (A) normal, (B) mild dysplasia, (C) carcinoma in situ (CIS), and (D) invasive squamous cell carcinoma (SCC) (original magnification, 200×). (E) White light image of the oral cancer at right side of tongue. Location of biopsies and its corresponding histology (A–D) are indicated. (F) Fluorescence visualization (FV) image of the same lesion, with a broad area of dark brown change in FV loss. (G) Alignment of genomic alteration data at chromosome 9p indicates 9p13 amplification in the CIS and SCC cells obtained from this single patient. The red vertical lines indicate log2 signal intensity ratios of +1 and +0.5 and the green lines indicate ratios of −1 and −0.5. Each black dot represents the log2 signal intensity ratio of an individual clone mapping to the array. (H) Increased fold-change mRNA expressions (>twofold) are found for STOML2, VCP, and DCTN3 as determined by gene expression microarray. No standard error is indicated for STOML2 as only a single probe maps to this gene.

Oral tumors have been known to exhibit a high degree of molecular heterogeneity 29–31. Genomic profiling and the detection of shared DNA alteration boundaries have been used to delineate clonal relationships and identify stage-specific genetic alterations for a variety of cancer types 29,32–34. To evaluate whether 9p13 DNA dosage changes lead to overexpression of our three candidate oncogenes during oral cancer progression, we undertook molecular analyses of matched DNA and RNA taken from multiple lesions within an oral cancer field from a single oral cancer patient. This field of diseased oral tissue was defined using a fluorescence visualization (FV) device and histopathological review. DNA and RNA samples were extracted from microdissected tissues representing normal, mild dysplasia, *CIS*, and oral squamous cell carcinoma (OSCC) tissues within the defined area (Fig.6A–F) 15,35,36.

We detected the 9p13 DNA copy number increase in the *CIS* and OSCC samples, with both samples exhibiting the same DNA alteration boundary (suggesting a shared clonal origin) (Fig.6G). This same DNA alteration event was not detected for normal or dysplasia tissues isolated from the same disease field. We noted that no genetic alterations were present in the whole genome profiles of the normal and mild dysplasias—and that genomic instability at 9p13 was first observed in the *CIS* sample in this oral cancer field. Expression levels of the three 9p13.3 candidate genes were evaluated in RNA samples isolated from each biopsy from the disease field (Fig.6H). Relative to matched normal tissue, *DCTN3* was the only candidate showing ≥twofold overexpression in all stages of disease. *VCP* and *STOML2* were transcriptionally upregulated only in the *CIS* lesion and invasive tumor, but did not show a change in those samples without the 9p13 copy number increase. *VCP*, *DCTN3*, and *STOML2* all showed expression increases concomitant with the emergence of DNA copy number increases at 9p13.3.

### Frequency of 9p13 gain in various cancers

To determine the significance of 9p13 DNA gain in other cancer cell types, we evaluated 217 cell lines derived from several cancer types 37. We found 36 (16.6%) of the cell lines carried regions of genomic gain spanning part of chromosome 9p13, while 4 (1.8%) harbored high-level DNA amplification of this region. Those four lines with high-level amplification included: a ductal breast carcinoma line (BT-474), a tongue squamous cell carcinoma line (SCC-9), a melanoma line (WM-115), and an osteosarcoma line (MG-63). Of these four lines, only the amplicon in SCC-9 spanned all four oral cancer candidate genes defined by our analyses of oral cancer tissues (*VCP*, *STOML2*, and *DCTN3*). These results suggest that 9p13 gain is not specific to oral tumorigenesis but may also play a role in malignancy in other cancer types.

## Discussion

Loss of chromosome 9p has been reported as an early and frequent event in oral tumorigenesis, with selection for the loss of tumor suppressor *CDKN2A* (at 9p21.3) believed to be the driving force behind the emergence of this alteration 12,38. Loss of chromosome 9p has primarily been detected by microsatellite markers for LOH analysis, with reports suggesting 9p LOH frequencies ranging from 28% to 82% in oral precancers. Here, an examination of progressing low-grade OPLs reveals that DNA copy number gain at 9p13 is detected at a frequency of 62.5%, whereas deletions on 9p21-24 are only found in 25% of cases. This discrepancy could be explained by (1) differences in analytical techniques (with LOH analysis not typically performed at the 9p13 locus); (2) differences in sample cohort make-up; and (3) the fact that earlier reports often involved LOH analysis of only severe dysplasia and *CIS* lesions when delineating molecular events in OPLs (i.e., low-grade OPLs were not included in analyses or analyzed separately). When we evaluate only high-grade OPLs within our data, we also detect a higher frequency of loss on chromosome 9p21, providing insight into a possible sequence of molecular events occurring during disease progression (data publicly available at GSE9193) 4. Additionally, our TMA experiments show 43.5% of progressing LGOPLs possess a DNA copy number gain at 9p13, whereas this is much less frequent in the high-grade OPL cohort (7.6%). A comparison of the available patient demographic data (such as age, sex, smoking history, and site of cancer) indicate our patient population is similar to those assessed in previous studies analyzing 9p21 deletion in OPLs 5,6.

DNA copy number gain at chromosome 9p13 has been reported as a driver for other epithelial cancer types. The frequency of gain in this region varies widely depending on cancer type, from ∼8% to 75% 23,39,40. A study analyzing DNA copy number data across 11 types of cancer—including head and neck cancers—found increased DNA dosage at 9p13.3 to be significantly recurrent 41. When only considering the DNA copy number changes in head and neck cancer in this same sample set, gains at chromosome 9p13.3 were found to be one of the most significantly recurring regions of DNA gain 41. To the best of our knowledge, only one other group has fine-mapped recurring amplifications in this region to identify oncogene candidates. Kamradt et al. 23 reported a minimal region of alteration of ∼1.7 Mb in prostate cancers that spanned two candidate genes—*IL11-RA* and *DCTN3*. Recently, 9p13 DNA gain has been reported for oral tumors 42,43. Pickering et al. 43 found that 26% of OSCC tumors possessed a 9p amplification and suggested an oncogene may be found within this area. Furthermore, analysis of the cohort of head and neck tumors from TCGA Research Network (http://cancergenome.nih.gov) using cBioPortal for Cancer Genomics and the Integrative Genomics Viewer (IGV) indicate that 27% of oral cavity specific tumors have a copy number gain at 9p13.3 (based on analysis of data from tissue collected from the oral cavity, tongue, floor of mouth, buccal mucosa, and hard palate) 44,45. These findings agree with our results insofar as gain at 9p13 occurs at a lower frequency in later stage/invasive disease as compared to earlier stage OPLs that progress.

To identify oncogene candidates within the 9p13 region, we leveraged existing whole genome alteration data from OPLs and oral tumors and global gene expression data from oral tumors. Then we further honed this list by evaluating only those candidates previously implicated in malignant processes. *VCP*, *STOML2*, and *DCTN3* were identified by this approach. *VCP* (*p97*) is known to activate NF-*κ*B signaling, drive cell proliferation, and antiapoptotic messages, and has been described as upregulated in multiple cancer types 22. *STOML2* has been described as a positive regulator of cancer cell growth; its increased expression is associated with esophageal precancers and its expression has been reported as increasing with invasive disease stages in laryngeal squamous cell carcinomas 24,26. High expression of *STOML2* is also associated with poorer survival in gastric adenocarcinoma, and metastasis and poor survival in lung cancer 28,46. *DCTN3* (also referred to as *DCTN22* or *p22*) is a subunit of dynactin, which is a protein complex involved in a number of cell processes such as spindle formation, cytokinesis, chromosome movement, and nuclear positioning 47. *DCTN3* has been associated with progression and metastasis formation in breast cancer 48. Each of these genes represented a robust oncogene candidate for downstream functional analysis.

Significantly, we found that all three oncogene candidates contributed to malignant processes in oral cancer cells. Independent shRNA-mediated knockdown of each candidate in SCC-9, an OSCC cell line harboring a 9p13 amplicon, resulted in reduced proliferative abilities and reduced capacity for anchorage-independent growth (Fig.3). Furthermore, lentiviral-mediated overexpression of *VCP*, *DCTN3*, and *STOML2* in Cal-27 and POE9n-tert, OSCC and dysplasia cell lines with neutral DNA copy number at 9p13, enhanced both proliferation and anchorage independent growth (Fig.4). These functional studies plus reports detailing the oncogenic potential of each of the three candidate genes potentially indicate that parallel activation of *VCP*, *STOML2*, and *DCTN3* may be a critical event in governing OPL phenotypes. However, DNA amplification is typically thought to arise by selection for overexpression of a single oncogene 2,49, our data indicate that multiple oncogenes may be driving emergence of the 9p13 amplicon in early OPLs. This concept of parallel oncogene activation has previously been reported in ovarian, colorectal, and breast cancers 50–52. These experiments give a strong indication that these genes can affect tumorigencity of tumor cells in vitro, however, in order to gain a more in-depth understanding of the effects of the gene in vivo, further investigations using animal models are warranted.

To further elucidate the impact of 9p13 gain on gene expression, we analyzed the DNA dosage and gene expression status of our three putative oncogenes in a series of different staged OPLs captured from within a single disease field (as defined by FV) 15,35,36 (Fig.6). Normal, mild dysplasia, *CIS*, and invasive oral carcinoma tissues were studied. In this case, the first sign of genomic instability at 9p occurred within the *CIS* lesion. The shared breakpoint at the 9p13 gain in both the CIS and OSCC biopsy suggest a shared clonal relationship between these two lesions. We assumed shared clonality between all four biopsies, however, as oral cancer potentially arises due to field cancerization, it is a possibility that all of these lesions are not in fact clonally related (as the normal and dysplasia oral tissues did not harbor any DNA copy number changes therefore breakpoint mapping was not feasible). DNA dosage–mediated increase in RNA expression was apparent for all candidate genes. Furthermore, our results suggest that activation of candidate oncogenes is likely to be governed by additional events rather than a single DNA copy number gain event at 9p13: we detected *DCTN3* overexpression in dysplasia tissue in the absence of increased gene dosage. However, all three genes were overexpressed by the invasive disease stage and exhibited DNA copy number gain-mediated expression increases. These results align well with the other clinical and functional data we report, further supporting the conclusion that these genes play a role in oral cancer phenotypes. This is also supported by trends of 9p13 gain and increased gene expression of our candidate genes in the oral cavity–specific samples from TCGA data. Gain of the 9p13 region was present in 27% of TCGA cases and overexpression of at least one of our candidate genes was present in 29% of cases. For tumors with 9p13 gain (*n* = 47) that had both gene expression and DNA copy number data available, 70% had an increase in gene expression of at least one of our candidate genes, however, 12% of cases without 9p13 gain showed overexpression of at least one candidates, further supporting the critical role of DNA dosage changes in driving overexpression of our candidate oncogenes (while also suggesting the existence of alternative mechanisms for deregulation of these genes in a smaller subset of cases).

Based on results from the single case we have reported and publicly available oral cancer data, it appears that in addition to DNA copy number changes, other factors may regulate expression of our candidate genes during oral tumorigenesis. Activation of oncogenes by a variety of mechanisms has previously been demonstrated in other types of cancer and reinforces the importance of analysis of tumors on multiple platforms 53. The fact that we have observed the 9p13 gain as well as gene overexpression at the CIS stage and not the matched dysplastic lesion in our single patient case indicates that there are multiple molecular pathways that can initiate oral tumorigenesis and that while the 9p13 gain event is one of the most highly frequent events within the progressing LGOPLs, it may also contribute to tumorigenesis at later stages as well.

The decrease in frequency of the 9p13 amplicon as disease progresses potentially indicates that this could be an initial step in disease initiation that is later not required or that other mechanisms of gene regulation other than DNA amplification are involved in maintaining gene overexpression. This decrease in frequency with increasing oral lesion severity may be explained by the increased genomic instability that is known to occur with disease progression. Tumor progression is a dynamic process, and thus early critical events for progression may lose selection pressure in favor of other molecular aberrations that confer a greater growth advantage 54. Known mechanisms of genome instability such as bridge fusion breakage can utilize a DNA amplification event to drive further fragility of the surrounding chromosome region, thus creating greater genome instability and driving possible loss of the original amplified region 55. This increased instability may be masking early genomic alterations that are important for disease initiation 56. Although there is a decrease in frequency of the 9p13 gain with disease progression, there is still a considerable proportion of tumors (26–27%) exhibiting this alteration and/or gene expression increases at invasive stages (as is evident from TCGA data and previous studies done on OSCC) 43.

## Conclusion

We report a recurrent region of DNA gain at 9p13 that is frequently detected in low-grade OPLs that subsequently progressed to invasive disease. Within this region, *VCP*, *STOML2*, and *DCTN3* were identified as candidate oncogenes and we demonstrate that each is able to regulate oral cancer phenotypes. Activation of these putative oncogenes is frequently mediated by this single DNA copy number gain event at chromosome 9p13. Analysis of multiple biopsies from a single oral cancer field suggests that additional levels of molecular dysregulation might govern activation of these genes, indicating that their contribution to oral cancer progression is more complex than a simple additive effect. Further analysis of this 9p13 alteration event in oral cancer initiation and progression is warranted.

## Supporting Information

**Table S1.** Patient demographic information for tissue microarray analysis.

**Table S2.** Relative gene expression data for each candidate gene in SCC-9, Cal27, DOK and POE9n-tert cell lines.
