# Supplementary material for: Recurring DNA copy number gain at chromosome 9p13 plays a role in the activation of multiple candidate oncogenes in progressing oral premalignant lesions
Source: Cancer Med. 2014 Jul 24;3(5):1170–84. doi: 10.1002/cam4.307 (PMC4302668; doi:10.1002/cam4.307)
Supplement: Supplementary file 3 — Table S2. Relative gene expression data for each candidate gene in SCC-9, Cal27, DOK and POE9n-tert cell lines. [file cam40003-1170-SD3.docx]

Table S1. Patient demographic information for tissue microarray analysis.

13

Table S2. Relative gene expression data for each candidate gene in SCC-9, Cal27, DOK and POE9n-tert cell lines.
